# Supplementation of Silymarin Alone or in Combination with Salvianolic Acids B and Puerarin Regulates Gut Microbiota and Its Metabolism to Improve High-Fat Diet-Induced NAFLD in Mice

**DOI:** 10.3390/nu16081169

**Published:** 2024-04-14

**Authors:** Xin Wang, Yufeng Jin, Can Di, Yupeng Zeng, Yuqing Zhou, Yu Chen, Zhijun Pan, Zhongxia Li, Wenhua Ling

**Affiliations:** 1 Department of Nutrition, School of Public Health, Sun Yat-sen University (Northern Campus), Guangzhou 510080, China; wangx523@mail2.sysu.edu.cn (X.W.); jinyf6@mail2.sysu.edu.cn (Y.J.); zengyp27@mail2.sysu.edu.cn (Y.Z.); zhouyq79@mail2.sysu.edu.cn (Y.Z.); cheny2255@mail2.sysu.edu.cn (Y.C.); panzhj5@mail2.sysu.edu.cn (Z.P.); 2 Guangdong Provincial Key Laboratory of Food, Nutrition and Health, Guangzhou 510080, China; 3 BYHEALTH Institute of Nutrition and Health, Guangzhou 510663, China; dican@mail3.sysu.edu.cncv

**Keywords:** dietary silymarin intervention, NAFLD, gut microbiota, fecal microbiome transplantation (FMT), bile acids, secondary bile acids, metabolomics analysis

## Abstract

Silymarin, salvianolic acids B, and puerarin were considered healthy food agents with tremendous potential to ameliorate non-alcoholic fatty liver disease (NAFLD). However, the mechanisms by which they interact with gut microbiota to exert benefits are largely unknown. After 8 weeks of NAFLD modeling, C57BL/6J mice were randomly divided into five groups and fed a normal diet, high-fat diet (HFD), or HFD supplemented with a medium or high dose of Silybum marianum extract contained silymarin or polyherbal extract contained silymarin, salvianolic acids B, and puerarin for 16 weeks, respectively. The untargeted metabolomics and 16S rRNA sequencing were used for molecular mechanisms exploration. The intervention of silymarin and polyherbal extract significantly improved liver steatosis and recovered liver function in the mice, accompanied by an increase in probiotics like *Akkermansia* and *Blautia*, and suppressed *Clostridium*, which related to changes in the bile acids profile in feces and serum. Fecal microbiome transplantation confirmed that this alteration of microbiota and its metabolites were responsible for the improvement in NAFLD. The present study substantiated that alterations of the gut microbiota upon silymarin and polyherbal extract intervention have beneficial effects on HFD-induced hepatic steatosis and suggested the pivotal role of gut microbiota and its metabolites in the amelioration of NAFLD.

## 1. Introduction

Non-alcoholic fatty liver disease (NAFLD), considered highly correlated with metabolic syndrome [1,2], is widely prevalent globally [3,4]. Due to the potential of its development into life-threatening chronic liver disease and other metabolic diseases, it has caused a huge economic burden [5,6,7]. In addition to abnormal liver metabolism promoting fatty liver, intestinal dysbiosis reportedly contributes to the severity of NAFLD, showing associations with altered gut microbiota and microbial metabolome [8,9,10]. The underlying mechanisms mainly include disruption of tight junctions and heightened gut permeability, translocation of lipopolysaccharide (LPS) and inflammatory mediators, decreased short-chain fatty acids, increased ethanol production, and changes in bile acids (BAs) and amino acid-derived metabolism [11,12].

Numerous phytochemicals in nature have poor bioavailability [13]. These inadequately absorbed constituents undergo substantial interaction with the intestinal microbiota upon ingress into the intestinal tract, which consequently possess the potential to confer health advantages by modulating and restructuring the gut microbiota [14,15,16]. Our antecedent investigations elucidate that polyphenolic compound resveratrol exhibits the capacity to modulate the intestinal microbiota, and results in the attenuation of microbiota-driven synthesis of secondary BAs within the murine intestinal, which finally mitigates the unwarranted absorption of dietary fats [17]. Furthermore, studies suggests that the dietary supplementation of plant-derived resistant starch can reshape the gut microbiota, altering the composition of BAs and the biological metabolism of amino acids, with ultimately alleviated hepatic lipid deposition and inflammation [18].

To summarize, modulating the intestinal microbiota and its metabolism through dietary intake of phytochemicals represents a potentially significant and readily accessible approach for treating NAFLD, as it lacks approved clinical therapeutic drugs, with current treatment strategies relying on dietary and lifestyle modifications [19,20].

Silymarin, a flavonolignan compound derived from the herbal plant Silybum marianum, demonstrates diverse hepatoprotective properties, encompassing antioxidative and hypolipidemic effects, and is characterized by low bioavailability [21,22,23]. Silybin is identified as its principal active constituent [22,23]. Accumulating evidence suggests that silymarin ameliorates the progression of NAFLD in both patients and experimental animals [24,25,26,27], and can reshape the composition of gut microbiota [28,29,30,31]. Research reported that silybin intervention reshaped the microbial community, along with an enrichment of short-chain fatty acids (SCFAs) and a decrease in secondary BAs in the gut [29]. Another study found significant changes in the microbiota and bacterial Vitamin B12 production of rats along with NAFLD amelioration following silymarin intervention [30].

Salvianolic acid B (Sal B) and puerarin, also as the active components extracted from traditional herbal medicines in Asia, have been extensively studied for their protective effects on metabolic homeostasis. Studies observed that Sal B could improve liver enzyme levels and regulate hepatic lipid metabolism in NAFLD mice [32]. Puerarin was observed to alleviate hepatic steatosis and metabolic disorders in rats [33]. It is noteworthy that silymarin, in combination with certain herbal ingredients, can significantly ameliorate the clinical symptoms of patients with NAFLD, while also improving lipid levels and liver function [34]. However, it is still unclear whether silymarin, in combination with these two active components, can collaboratively ameliorate NAFLD by modulating the gut microbiota.

Due to the limited insight into the mechanistic actions of silymarin on the functionality of the gut microbiota and its generated metabolites, we conducted a Silybum marianum extract (silymarin) and polyherbal extract (silymarin, Sal B, puerarin) intervention experiment using a high-fat-diet-induced NAFLD mouse model. The intervention increased probiotics such as *Akkermansia* and *Blautia* and suppressed the genera related to secondary BAs biosynthesis, along with enriching SCFAs and inhibiting secondary BAs in the gut. Results from fecal microbiome transplantation (FMT) confirmed that the alteration of microbiota and its metabolites was a crucial link in the effect that silymarin and polyherbal extract had in reducing hepatic lipid accumulation, enhancing liver function, and improving NAFLD.

## 2. Materials and Methods

### 2.1. Materials

Silybum marianum extract contained silybin and polyherbal extract (Silybum marianum extract, Pueraria root extract, Salvia miltiorrhiza extract, and schisandra extract) contained silybin, Sal B, and puerarin were provided by BYHEALTH Co., Ltd. (Guangzhou, China), and were added to a high-fat diet (HFD; 45% energy from fat, 20% from protein and 35% from carbohydrate; MD12032, Medicience, Yangzhou, Jiangsu, China) for intervention. Specifically, under the sterile laminar flow hood, 45% high-fat powdered feed was incorporated with an intervention substance or left without an intervention substance, followed by thorough homogenization through stirring. Subsequently, the diet was pelletized through compression molding and stored at −20 °C until utilization. Table 1 presents the specific substance content in intervention feeds.

### 2.2. Animal Models and Experiment Design

Seven-week-old male C57BL/6J mice were purchased from the Experimental Animal Center of Guangdong Province (Guangzhou, China) and maintained in a specific pathogen-free facility under a 12 h dark/light circle at 25 ± 0.5 °C and 50–60% humidity (five mice per cage). After a one-week acclimatization period, mice were randomly divided into two groups. The mice in the model group were fed with 45% HFD to induce NAFLD while the normal control mice (NC group, *n* = 10) were fed with a normal chow diet (4.2% crude fat, MD17121, Jiangsu Medicience, Yangchow, China). After the successful eight-week modeling, NAFLD mice were randomly divided into four groups: (1) The HF mice continued to be fed 45% HFD as previously. (2) The MSL mice were fed an HFD supplemented with a medium dose of silymarin. (3) The HSL mice were fed an HFD supplemented with a high dose of silymarin. (4) The PD mice received HFD supplemented with polyherbal extract. These groups, along with the NC group, participated in the subsequent silymarin intervention experiment for 16 weeks (*n* = 10 mice/group).

Our intervention doses were determined based on reported animal experiments and converted according to the dietary intake of 0.1 g/g·bw·day. Mice were given free access to food and water, and their body weight was recorded weekly. In the last week of the experiment, an adequate amount of feces was collected from the HF mice, HSL mice, and PD mice, frozen in liquid nitrogen, and stored at −80 °C for fecal microbiota transplantation (FMT) experiments. After 16 weeks of dietary intervention, all mice were subjected to an overnight fast. Subsequently, they were anesthetized (1% pentobarbital, 0.01 mL/g) for blood collection from the eye sockets and euthanized by cervical dislocation. Serum samples were obtained by centrifuging blood at 3000 rpm for 10 min and stored at −80 °C. Additionally, part of their liver samples were fixed in 4% paraformaldehyde (F8775, Sigma-Aldrich, Hartford, CT, USA) for histological analysis, while the remaining samples were immediately frozen in liquid nitrogen and stored at −80 °C. All procedures were approved and permitted by the Institutional Review Boards and Animal Care and Use Committees of Sun Yat-Sen University.

### 2.3. Fecal Microbiome Transplantation (FMT)

The FMT experiment was executed adhering to a well-established protocol, with slight modifications incorporated for optimization [35]. Fecal samples (100 mg) were resuscitated in a water bath at 37 °C for 20 min. Afterward, the samples were re-suspended in 1 mL PBS, thoroughly mixed, and then centrifuged at 1000 rpm for 5 min. Filtered through the 100 μm filter, the transplantation material was obtained. The above preparation was conducted within 30 min before each FMT experiment.

C57BL/6J mice were randomly assigned to the control group (ONC, *n* = 8) and the model group. After inducing NAFLD for 8 weeks using the 45% HFD, NAFLD mice were further randomized into OHF, OHSL, and OPD groups (*n* = 8 mice/group). All mice received an oral gavage of broad-spectrum antibiotics for 4 weeks (every three days) to establish a pseudo-germ-free model, according to a validated experimental methodology. Starting from the 12th week, OHF, OHSL, and OPD groups received transplant materials from HF, HSL, and PD groups, respectively, while the ONC group underwent 10% PBS. FMT experiments were performed every three days, totaling 12 weeks. At the end of the experiment, mice were anesthetized (1% pentobarbital, 0.01 mL/g) for blood collection from the eye sockets and sacrificed by cervical dislocation. Serum samples were obtained by centrifuging blood at 3000 rpm for 10 min, and stored at −80 °C. Part of their liver samples were fixed in 4% paraformaldehyde (F8775, Sigma-Aldrich, USA) for subsequent histological analysis, while the remaining samples were immediately frozen in liquid nitrogen and stored at −80 °C. All efforts were made to minimize animal suffering and reduce the number of animals used.

### 2.4. Intraperitoneal Glucose Tolerance Test (IGTT) and Intraperitoneal Insulin Tolerance Test (IPITT)

Eight mice were randomly selected to perform IGTT and IPITT from each group two weeks and one week before the end of the intervention, respectively. After an 8-hour fasting period, the body weight and level of fasting blood glucose of the mice were measured. Following that, mice were subjected to intraperitoneal injection of glucose solution (2 g/kg·bw, G885129, Macklin, Shanghai, China) or insulin solution (0.75 U/kg·bw, PB180432, Pricella, Wuhan, China). Blood samples were collected from the tail vein at 15, 30, 60, and 120 min post injection, and glucose levels were immediately measured using the blood glucose meter (GA-3, Sinocare, Changsha, China). Glucose tolerance and insulin tolerance for each group were assessed by calculating the area under the curve (AUC) of the blood glucose levels over the specified time intervals.

### 2.5. Biochemical Analysis

The levels of triglycerides (TG), total cholesterol (TC), tumor necrosis factor-alpha (TNF-α), and interleukin-17 (IL-17) in liver tissue were determined using ELISA kits (JL11109; JL-T1371; JL10484; JL20250; Jianglai, Shanghai, China) following the manufacturer’s instructions. Levels of serum TC, TG, high-density lipoprotein cholesterol (HDL-C), low-density lipoprotein cholesterol (LDL-C), fasting blood glucose (FBG), alanine aminotransferase (ALT), and aspartate aminotransferase (AST) were measured by fully automated biochemical analyzer (V501536; V526234; V257033; V251528; V257024, BS-830, Mindray Bio-Medical Electronics, Shenzhen, China).

### 2.6. Histological Analysis

A portion of prefixed liver tissue in 4% formalin was removed, embedded in paraffin, and sectioned at a thickness of 5 μm. These sections were then stained with hematoxylin and eosin (H&E). Another portion was embedded in Tissue-Tek O.C.T. Compound (4583, SAKURA, Seattle, WA, USA), cryosectioned, and stained with Oil Red O (O1391, Sigma-Aldrich, USA). Examination, observation, and imaging were performed using the panoramic tissue cell quantification system (TissueFAXS Plus S, TissueGnostics, Vienna, Austria).

### 2.7. 16S rRNA Sequencing and Analysis

Fecal bacterial DNA extraction was performed utilizing the E.Z.N.A.^®^ Stool DNA Kit (D4015, OMEGA, Bargersville, IN, USA) following the manufacturer’s instructions, and the concentration and purity of DNA were assessed by micro spectrophotometer (NanoDrop™ One, Thermo Fisher Scientific, Waltham, MA, USA). The amplification of the V4 hypervariable regions of the 16S rRNA was achieved through PCR (95 °C for 3 min, followed by 27 cycles at 95 °C for 30 s, 55 °C for 30 s, and 72 °C for 45 s, with a single extension at 72 °C for 10 min, and concluding at 4 °C). Primers 515F (GTGCCAGCMGCCGCGGTAA) and 806R (GGACTACHVGGGTWTCTAAT) were employed for PCR, conducted using the BioRad S1000 PCR thermocycler (Bio-Rad Laboratory, Hercules, CA, USA). The PCR products were subjected to concentration normalization using GeneTools Analysis Software (Version 4.03.05.0, Syngene, Frederick, MD, USA) [36]. Library construction adhered to the standard procedure outlined in the NEBNext^®^ Ultra™ II DNA Library Prep Kit for Illumina^®^ (New England Biolabs, Ipswich, MA, USA). Subsequently, the amplified library was sequenced using the Illumina Nova 6000 platform (Magigene Biotechnology Co., Ltd., Guangzhou, China). The data concatenation and filtering processes were conducted using the fastp software (version 0.14.1, https://github.com/OpenGene/fastp, accessed on 7 March 2023), usearch-fastq_mergepairs (Version 10, http://www.drive5.com/usearch/, accessed on 7 March 2023), and cutadapt software (Version 4.2, https://github.com/marcelm/cutadapt/, accessed on 8 March 2023) [37]. Subsequently, the merged sequences underwent analysis using the QIIME software (Version 1.9.1) [38]. High-quality reads were clustered into operational taxonomic units (OTUs) based on 97% sequence similarity. Taxonomic annotations were assigned to representative sequences by aligning them in the SILVA 16S database (https://www.arb-silva.de/, accessed on 13 March 2023) [39], with a confidence threshold set at 0.8.

Alpha and beta diversity analyses were conducted using QIIME (Version 1.9.1) and visualized with R software (Version 2.15.3). The Bray–Curtis distance algorithm analysis was performed based on the OTU abundance table, and principal co-ordinates analysis (PCoA) was utilized for dimensionality reduction and visualization. The OTU table was normalized for differential abundance analysis using the Linear discriminant analysis Effect Size (LEfSe) online platform (http://huttenhower.sph.harvard.edu/galaxy, accessed on 21 March 2023) [40], and Linear Discriminant Analysis (LDA) was employed to achieve dimensionality reduction and assess the impact size of differentially significant species (LDA Score), with a filtering value set at 3.0. Standardization of the OTU abundance table was carried out using PICRUSt (Version 2.0) [41], and the Greengene ID corresponding to each OTU was aligned with the KEGG database to obtain pathway information. The abundance of various functional categories at different levels was calculated based on the OTU abundances. Non-parametric factorial Kruskal–Wallis sum rank test was employed to detect differences between different groups, while Wilcoxon rank-sum test was used for pairwise group comparisons.

### 2.8. Metabolites Extraction and Untargeted Metabolomics Analysis

An ultra performance liquid chromatography and tandem mass spectrometry (UPLC-MS/MS) analysis were conducted utilizing the Vanquish™ UHPLC system coupled with the Orbitrap Q Exactive™ HF-X mass spectrometer (Thermo Fisher Scientific, Freiburg, Baden-Württemberg, Germany). 100 μL serum samples and 100 mg homogenized feces were combined with 400 μL and 500 μL 80% methanol–water solution, respectively. Following vortexing, ice bath incubation, and centrifugation, the collected supernatant was diluted with mass spectrometry-grade water to achieve a methanol content of 53%. Subsequently, after centrifugation at 415,000× *g* and 4 °C for 20 min, the obtained supernatant underwent LC–MS analysis with instrument parameters set according to a standard protocol. Compound Discoverer software (Version 3.1) was utilized for post-sequencing raw data processing, and the obtained results underwent comparison with mzCloud databases (https://www.mzcloud.org/, accessed on 2 June 2023). After standardization, metabolite identification and relative quantification were achieved.

Metabolite annotation was carried out based on the KEGG database (https://www.genome.jp/kegg/pathway.html, accessed on 9 June 2023), HMDB database (https://hmdb.ca/metabolites, accessed on 9 June 2023), and the LIPID MAPS database (http://www.lipidmaps.org/, accessed on 9 June 2023). Following data transformation using metaX software (http://metax.genomics.cn/, accessed on 13 June 2023) [42], principal component analysis (PCA) and partial least squares discriminant analysis (PLS-DA) were performed, yielding variable importance in the projection (VIP) values for each metabolite. Statistical analysis involved Student’s *t*-test to calculate the *p* value of metabolites among two groups, along with the computation of fold changes (FC). The default criteria for selecting differential metabolites were set at VIP > 1, *p* < 0.05, and FC ≥ 2 or FC ≤ 0.5. Z-score normalization was applied to metabolite data, and the functional annotation of differed metabolites was explored based on KEGG database. Enrichment analysis of metabolic pathways was conducted based on the criteria x/*n* > y/*N* for pathway enrichment, and significance was determined at *p* < 0.05. Data processing was conducted using the Linux operating system (CentOS version 6.6) and Python (Version 3.5.0) while the visualization was performed by R software (Version 3.4.3).

### 2.9. Statistical Analysis

The experimental data were statistically analyzed and graphed using GraphPad Prism (Version 9.0, GraphPad Software, San Diego, CA, USA). Comparisons between two groups were performed using an unpaired two-tailed *t*-test or Mann–Whitney nonparametric test where appropriate, while one-way analysis of variance (ANOVA) followed by the Dunnett’s post hoc test was conducted for the comparison between multiple groups. Spearman correlation analysis was conducted using R (co(r), Version 3.4.3) and the results was visualized by corrplot package. Data were expressed as mean ± standard deviation (SD). Statistical details are included in the figure legends where ‘*n*’ represents biological replicates across all experiments. * *p* < 0.05; ** *p* < 0.01; *** *p*< 0.001; **** *p*< 0.001.

## 3. Results

### 3.1. Silymarin and Polyherbal Extract Attenuate HFD-Induced Steatohepatitis

**Figure 1:**
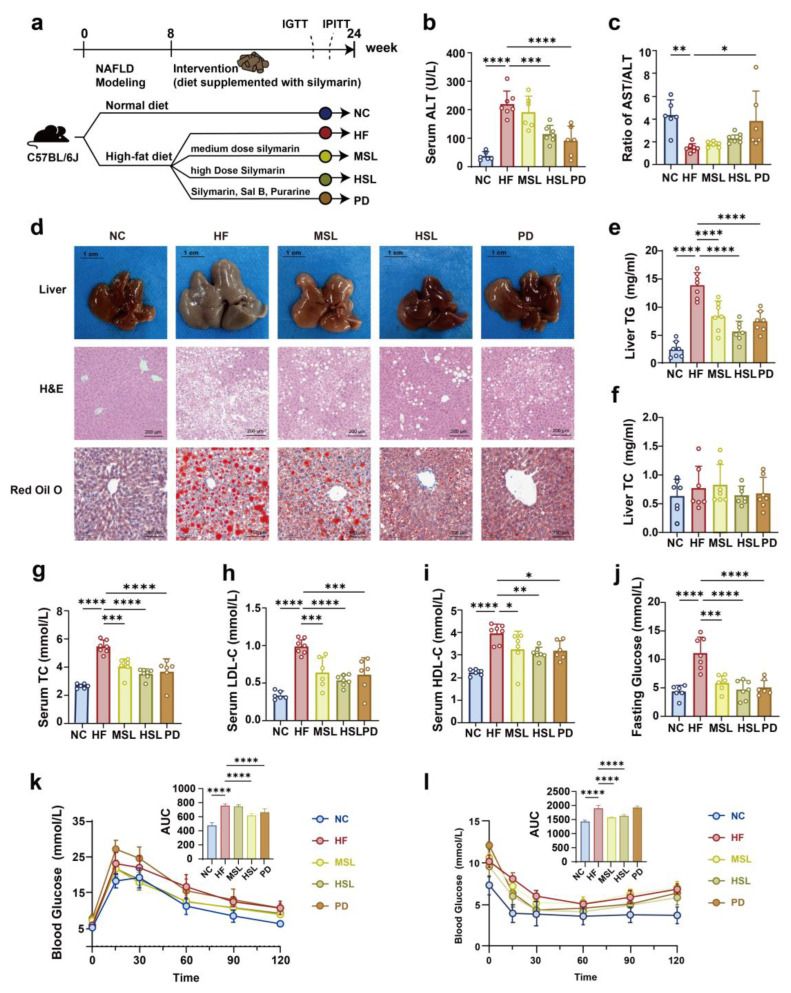
Effect of silymarin and polyherbal extract on the pathological and biochemical indexes of NAFLD in mice. (**a**) Experiment design of silymarin and polyherbal extract intervention. (**b**,**c**) Levels of serum ALT (**b**) and the ratio of serum AST to ALT (**c**). (**d**) Representative morphology (Scale bars, 1 cm), representative microphotograph of hematoxylin and eosin (H&E) staining (Scale bars, 200 µm) and Oil Red O (ORO) staining (Scale bars, 100 µm) of livers. (**e**,**f**) Levels of liver TG (**e**) and liver TC (**f**); *n* = 7. (**g**–**j**) Levels of serum TC (**g**), LDL-C (**h**), HDL-C (**i**) and fasting blood glucose (**j**); *n* = 6 for the NC, MSL and PD group and *n* = 7 for the HF and HSL group. (**k**,**l**) Blood glucose level and area under the curve (AUC) during IGTT (**k**) and IPITT (**l**); *n* = 8. Values were shown as mean ± SD. Statistical significance was evaluated by two-sided one-way ANOVA with Dunnett’s post hoc test (compared with HF group); * *p* < 0.05, ** *p* < 0.01, *** *p* < 0.001, **** *p* < 0.0001. ALT alanine transaminase, AST aspartate aminotransferase, TG triglycerides, TC total cholesterol, HDL-C high-density lipoprotein cholesterol, LDL-C low-density lipoprotein cholesterol, IGTT intraperitoneal glucose tolerance test, IPITT intraperitoneal insulin tolerance test.

**Figure A1:**
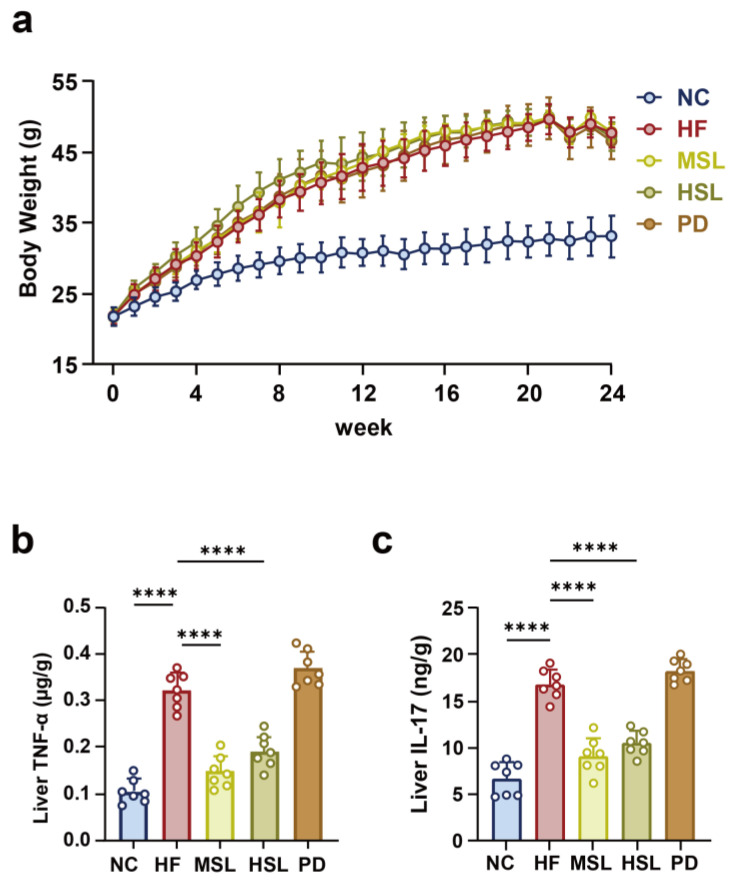
Effect of silymarin and polyherbal extract on the body weight and liver inflammatory indicators of NAFLD mice. (**a**) Body weight change in mice during 24 week intervention; *n* = 10 for the NC and MSL group and *n* = 11 for the HF, HSL and PD group. (**b**,**c**) Levels of liver TNF-α (**b**) and IL-17 (**c**) in all groups; *n* = 7. Values were shown as mean ± SD. Statistical significance was evaluated by two-sided one-way ANOVA with Dunnett’s post hoc test (compared with HF group); **** *p* < 0.0001. IL-17 interleukin 17, TNF-α tumor necrosis factor alpha.

NAFLD mice were induced by an HFD for 8 weeks, after which the mice were treated with silymarin or polyherbal extract for 16 weeks (Figure 1a). The body weight of mice with an HFD increased significantly compared to the control group, which showed no significant difference by silymarin or polyherbal extract intervention (Figure A1a). To evaluate the effect of the indicated treatment on liver function, we examined whether an HFD caused severe liver function injury, as indicated by an increase in the serum ALT and a decrease in the serum AST/ALT ratio. Treatment with silymarin, especially for the polyherbal extract intervention, restored liver function injury (Figure 1b,c) and this hepatoprotective effect of silymarin was further tested by histologic evaluations. Dietary silymarin and polyherbal extract supplement alleviated the hypertrophy and graying of liver morphology in contrast with the NAFLD mice. H&E and oil red O staining of livers showed that silymarin could effectively reduce the serious accumulation of liver lipid droplet and the extent of hepatocyte ballooning degeneration of the liver (Figure 1d). Additionally, results from hepatic TG, hepatic TC (Figure 1e,f), serum TC, HDL, and LDL (Figure 1g–i) showed that silymarin and the polyherbal extract had restored lipid metabolism disorders in the HF group. Further, the fasting glucose was significantly increased in the HF group and was rescued by silymarin and polyherbal extract (Figure 1j). Moreover, the silymarin-treated group had an improved glucose tolerance (Figure 1k) and insulin tolerance (Figure 1l). The pro-inflammatory cytokines tumor necrosis factor-α (TNF-α) and interleukin-17 (IL-17) in the liver were also decreased after silymarin intervention (Figure A1b,c). These observations suggested comparable improvement in HFD-induced liver damages upon silymarin and polyherbal extract supplement.

### 3.2. Silymarin and Polyherbal Extract Modulated-Flora Are Associated with Improvement in Steatohepatitis

In light of the fact that silymarin is characterized by notably low bioavailability, which suggests its effective interaction with the intestinal microbiota, and with the prevailing recognition in the association between gut microbiota and NAFLD, it could be suggested that silymarin intervention induced benefits that might be derived from the alteration of intestinal microbiota. We thus employed 16S rRNA gene sequencing on fecal samples to examine whether silymarin and polyherbal extract intervention might result in microbiota that potentially exert the effects of ameliorating NAFLD.

**Figure 2:**
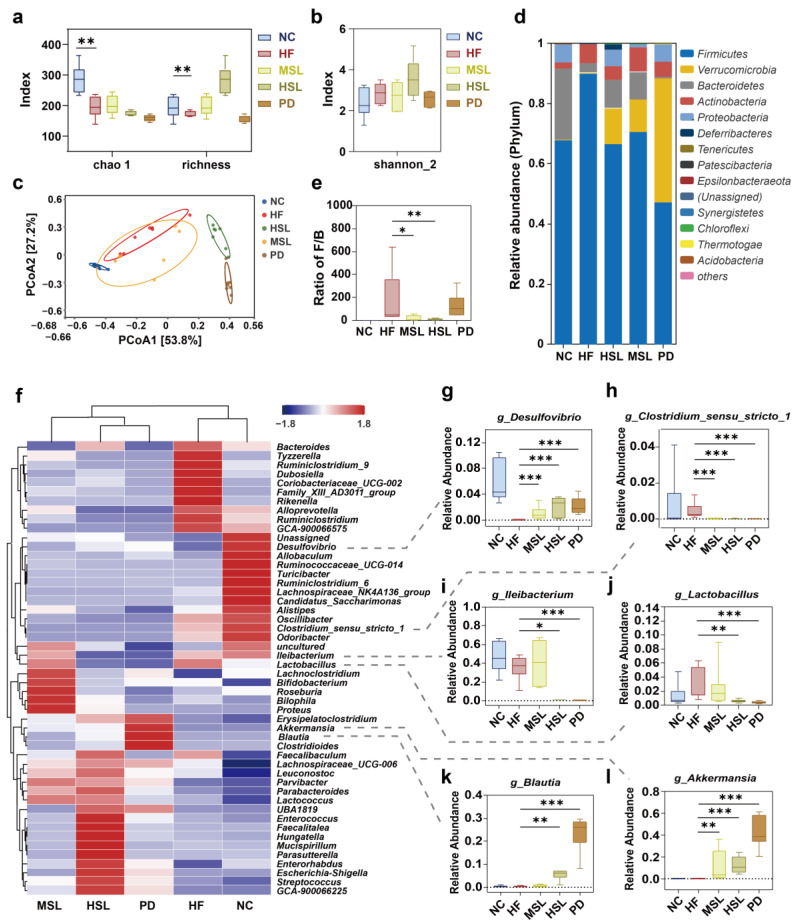
Silymarin and polyherbal extract modulates the composition of gut microbiota. 16S rRNA gene sequencing analysis in fecal bacterial DNA from NC, HF, MSL, HSL, and PD mice was performed; *n* = 7 individuals/group. (**a**,**b**) Alpha diversity was assessed by chao 1, observed richness (**a**) and Shannon_2 diversity index (**b**), respectively. (**c**) Bray–Curtis beta diversity was visualized with the principal coordinate analysis (PCoA). (**d**,**e**) The stacking histogram showing the taxonomic summary of phyla composition in feces from all groups (**d**) and the boxplots showing the ratio of fecal Firmicutes to Bacteroidetes in relative abundance (**e**). (**f**) The heatmap shows the relative abundance clustering (average) of microbial communities at the genus level in all groups. (**g**–**l**) The boxplots show statistical differences of selected differentially abundant genus between groups. Two-sided Mann–Whitney nonparametric test were conducted for comparisons; * *p* < 0.05, ** *p* < 0.01, *** *p* < 0.001. The horizontal line in each box represents the median, the top and the bottom of the box the 25th and 75th percentiles, and the whiskers the min to max.

The alpha diversities of Chao1, Richness, and Shannon_2 (Figure 2a,b) were comparable with intra-individual variance in the HF group and treated groups. However, the Bray–Curtis principal coordinate analysis (Figure 2c) manifested that the operational taxonomic units (OTUs) were clearly separated into four isolated groups, confirming an altered gut microbiota composition upon silymarin and polyherbal extract intervention. The relative abundance analysis revealed that, at the phylum level (Figure 2d), the treated group had a decreased *Firmicutes* along with an increased *Verrucomicrobia* and *Bacteroidetes*, and the HFD-induced high ratio of *Firmicutes*/*Bacteroidetes* was decreased in silymarin supplemented groups (Figure 2e). In other studies, these alterations were considered related to NAFLD improvement [43,44]. As Figure 2f displays, at the genus level, the abundance of *Costridium_sensu_stricto_1*, *Ileibacterium*, and *Lactobacillus*, which were encouraged by an HFD, reduced significantly in the HSL group, while the abundance of *Desulfovibrio*, *Blutia*, and *Akkermansia* increased compared with the HF group. In addition, this tendency of variation had a greater magnitude upon polyherbal extract intervention (Figure 2g–l). To sum up, silymarin and polyherbal extract treatment repressed HFD-induced microbiota, especially *Clostridium* and *Ileibacterium*, and benefited some probiotics such as *Akkermansia*.

**Figure 3:**
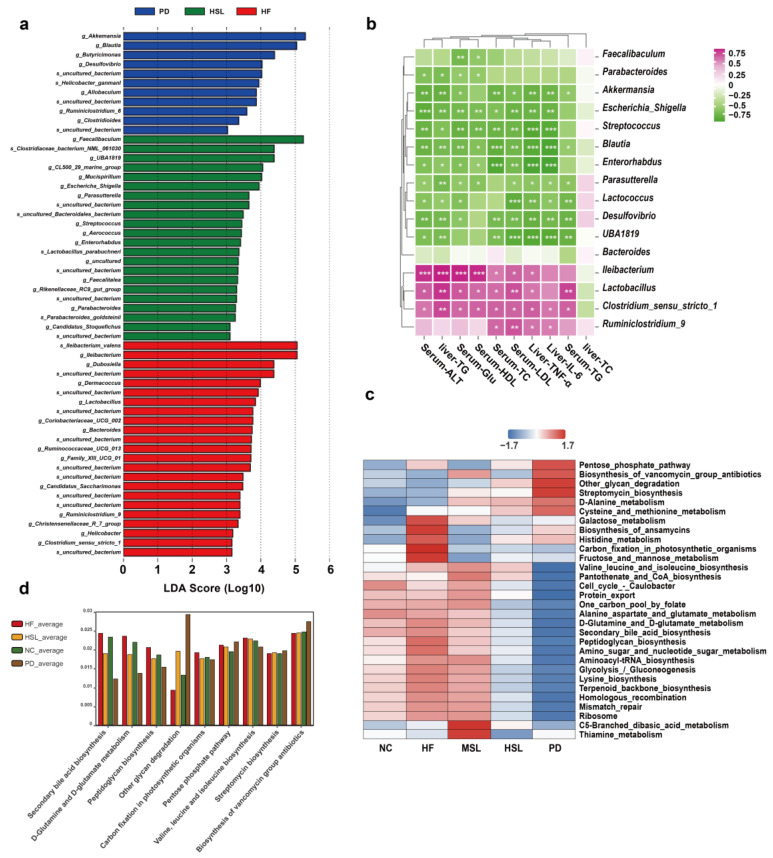
Silymarin and polyherbal extract modulated microbiota are related to NAFLD improvement and secondary bile acid biosynthesis. (**a**) Histogram of LDA score generated by LEfSe depicting the taxonomic contribution between microbiome communities from HF, HSL and PD mice (LDA > 3.0); *n* = 7. (**b**) Heatmap shows the correlations between the selected differentially abundant genus and various indicators related to NAFLD in HF and HSL groups; *n* = 6 and the correlations were analyzed using two-sided Spearman’s correlation, FDR-adjusted *p* < 0.05 (*, **, and *** indicate adjusted *p* < 0.05, 0.01, and 0.001, respectively) was shown. (**c**) Heatmap of KEGG functional pathway clustering (average) analysis reflects the functional composition between five groups based on OTU abundance. (**d**) The KEGG pathway with significant differences (adjusted *p* < 0.05) analyzed by Kruskal-Wallis H test among NC, HF, HSL and PD mice was shown; *n* = 7. LDA linear discriminant analysis, FDR false discovery rate, KEGG Kyoto Encyclopedia of Genes and Genomes.

To further investigate whether the above alteration of gut microflora might be associated with the amelioration of NAFLD, we first performed a linear discriminant analysis effect size analysis (LEfSe) and found a marked predominance in *Akkermansia*, *Blutia*, *Butyricimonas*, *faecalibaculum* of the HSL and PD group, while *Ileibacterium*, *Lactobacillus*, *Bacteroides*, *Costridium_sensu_stricto_1* was the core genus of the HF group (LDA score > 3) (Figure 3a). In a subsequent correlation test, the level of liver TG, serum ALT, and other indicators of liver inflammation and lipid metabolism presented marked positive correlation with *Ileibacterium*, *Lactobacillus*, *Clostridium_sensu_stricto_1*, and were significantly negatively correlated with changed *Akkermansia*, *Blutia* (Figure 3b). Additionally, from a KEGG functional pathway analysis based on OTU abundance (Figure 3c,d), we found that the altered floras were especially related to the suppressed secondary bile acid biosynthesis pathway (*p* = 0.00035) following intervention. These results suggested that the altered microbiota by silymarin and polyherbal extract might indeed relate to the amelioration of NAFLD, and the benefits might be achieved through metabolic products of the altered microbiota, particularly the BAs.

### 3.3. Transplantation of Altered-Microflora Ameliorate NAFLD

**Figure 4:**
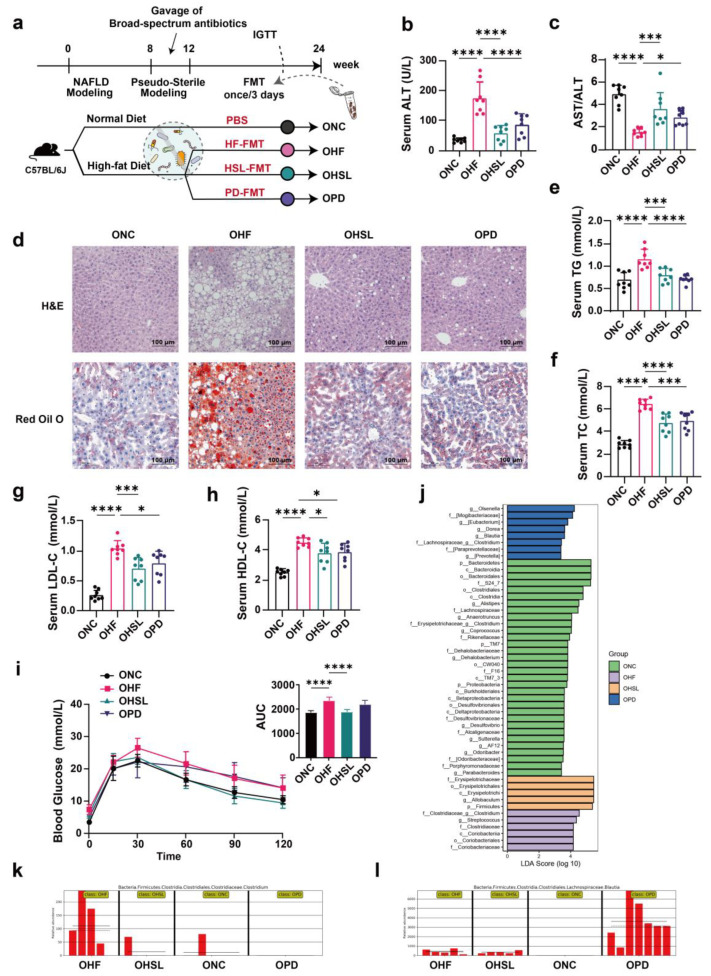
Alterations of liver pathobiology, serum biochemistry, and intestinal microbiome in NAFLD mice following FMT. (**a**) Experiment design of FMT; C57BL/6J mice were randomized into 4 groups (ONC, OHF, OHSL and OPD; *n* = 10); ONC group were under a control diet and others were fed with HFD (45% fat) throughout the trial period. After 8 weeks of NAFLD modeling, all groups were put on a course of intragastric broad-spectrum antibiotics administration for 4 weeks, and then OHF, OHSL, and OPD mice were colonized with HF, HSL, and PD mice derived fecal samples for 12 weeks, respectively. (**b**,**c**) Serum ALT (**b**) and the ratio of serum AST to ALT (**c**) after FMT; *n* = 7. (**d**) Representative photomicrographs of fixed liver sections after staining with H&E (Scale Bars, 100 μm) and ORO (Scale Bars, 100 μm). (**e**–**h**) Levels of serum TG (**e**), TC (**f**), LDL-C (**g**), HDL-C (**h**); *n* = 7. (**i**) Blood glucose level and area under the curve (AUC) during IGTT; *n* = 8. (**j**–**l**) The fecal from FMT mice were also collected for 16S rRNA gene sequencing analysis (V3–V4 region); *n* = 7 in NC, PD group and *n* = 5 in HF, HSL group. Histogram depicting the taxonomic contribution determined by LDA score (LDA > 2.5) under LEfSe (**j**) and changes in the genera of *Clostridium* (**k**) and *Bluatia* (**l**) with significant differences between groups was shown. Data were represented as mean ± SD. Statistical significance was evaluated by two-sided one-way ANOVA with Dunnett’s post hoc test (compared with OHF group); * *p* < 0.05, *** *p* < 0.001, **** *p* < 0.0001.

To verify the participation of intestinal microbiota and its metabolites in the amelioration of NAFLD upon silymarin and polyherbal extract treatment, we conducted fecal microbiota transplantation (FMT) experiments. Fecal microbiota from HF, HSL and PD mice were transferred into three additional groups of HFD-induced NAFLD mice, respectively (Figure 4a). After 12 weeks of FMT, we observed a reduced level of serum ALT and an increased ratio of AST to ALT in HSL-FMT and PD-FMT mice (Figure 4b,c), consistent with the liver function recovery in intervention experiments, paralleling with significantly reduced extent of obviously visible lipid droplets accumulation and hepatocyte ballooning degeneration in the liver tissue of H&E and ORO staining (Figure 4d). Further, silymarin and polyherbal extract FMT likewise led to lower levels of serum TG, TC, LDL, HDL (Figure 4e–h). In addition, an improved glucose tolerance was observed in HSL-FMT mice (Figure 4i).

**Figure A2:**
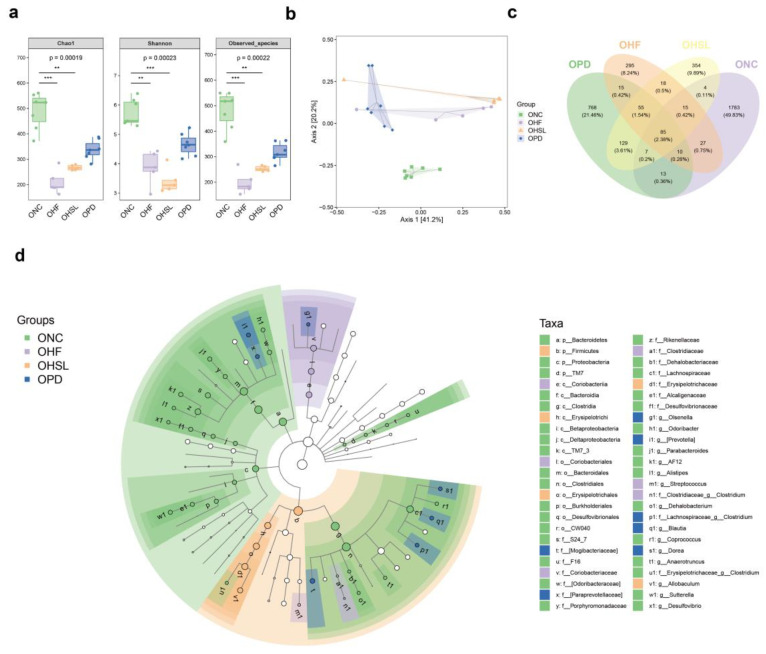
Alterations of intestinal microbiome in NAFLD mice following FMT. Results from 16S rRNA gene sequencing analysis (V3–V4 region) in the fecal of FMT mice; *n* = 7 in NC, PD group and *n* = 5 in HF, HSL group. (**a**,**b**) Alpha diversity was assessed by chao 1 index, Shannon diversity index and observed species, respectively (**a**), and unweighted-UniFrac beta diversity was visualized with the PCoA (**b**). (**c**) Venn diagram illustrates the number of common and unique microbiota between different comparison pairs. (**d**) Cladograms generated by LEfSe depicting taxonomic association between microbiome communities from NC, HF, HSL and PD mice. ** *p* < 0.01, *** *p* < 0.001.

Consistent with the results in the silymarin intervention, although the alpha diversity in intestinal flora of mice undergoing FMT observed without significance (Figure A2a), the composition of microflora was relatively different among groups (Figure A2b). In addition, HSL-FMT and PD-FMT also restored the reduction in characteristic bacteria induced by HF-FMT (Figure A2c). The LDA score (Figure 4j) generated by LEfSe (Figure A2d) revealed a marked predominance in the bacterial genera *Allobaculum* of HSL-FMT mice, *Blautia* of PD-FMT mice, and *Clostridium* of HF-FMT mice. Importantly, the HFD-induced *Clostridium* (Figure 4k) was likewise repressed while *Blautia* (Figure 4l) was encouraged by HSL-FMT and PD-FMT, in agreement with our previous finding. The above data demonstrated that the colonizing of altered microbiota from silymarin and polyherbal extract treated mice directly ameliorated NAFLD, further supporting that gut microbiota and its metabolites played a pivotal role in the NAFLD improvement in response to silymarin and polyherbal extract intervention.

### 3.4. Silymarin and Polyherbal Extract Regulate Fecal Metabolic Profiles and Inhibit the Biosynthesis of Secondary Bile Acids

**Figure 5:**
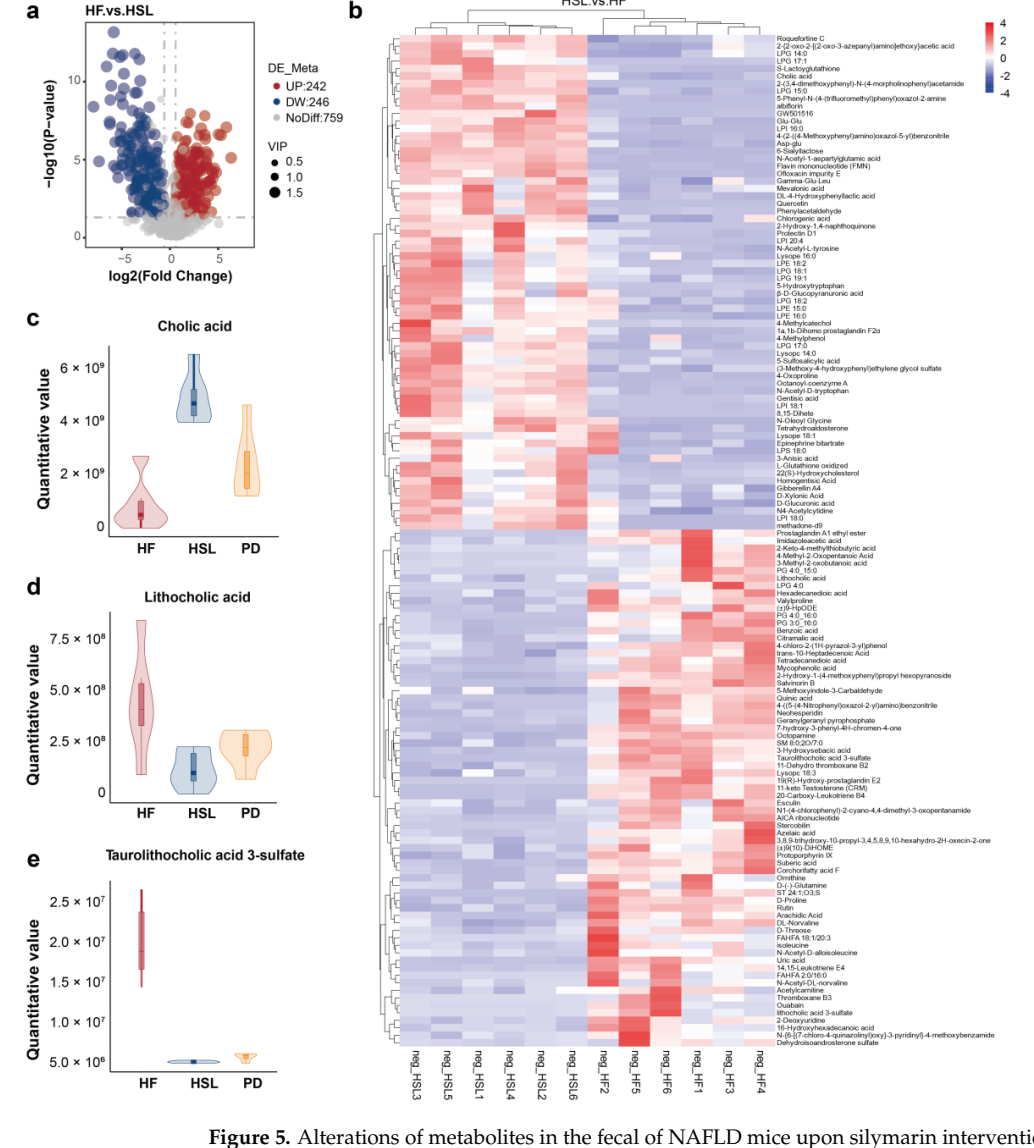
Alterations of metabolites in the fecal of NAFLD mice upon silymarin intervention. (**a**) The number of significantly changed metabolites in the feces (determined by Log_2_ FC and −Log_10_ *p* value) of HF mice compared to HSL mice was shown in the Volcano plot. (**b**) Heatmap showing fecal metabolites differed significantly in abundance between HF group and HSL group, blocks in red and blue denote high and low FC values, respectively. (**c**–**e**) Violin plots shows the significantly differed quantitative value of CA (**c**), LCA (**d**), and TLCA-sulfate (**e**) in HF, HSL, and PD group; *n* = 6. Significantly different metabolites were screened out by PLS-DA model (VIP > 1 and *p* < 0.05). FC Fold Change, CA cholic acid, LCA lithocholic acid, TLCA-sulfate taurolithocholic acid-sulfate, PLS-DA partial least squares-discriminant analysis, VIP variable importance for the projection.

**Figure A3:**
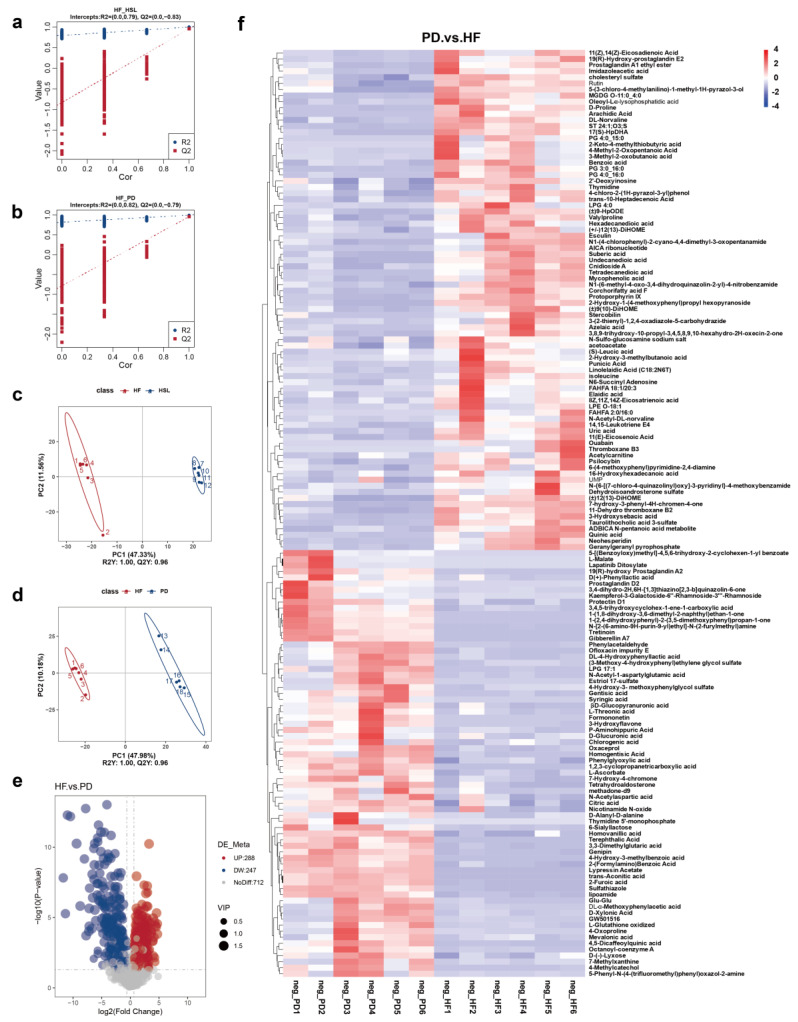
Alterations of metabolites in the fecal of NAFLD mice upon polyherbal extract intervention. Analysis results of non-targeted metabolomics in the feces from HF, HSL, and PD mice; *n* = 6. Permutation tests conducted with 999 random per mutations in the PLSDA model of HF vs. HSL group (**a**) and HF vs. PD group (**b**). (**c**,**d**) Principal component analysis (PCA) plot. (**e**) The number of significantly changed metabolites in the feces of HF mice compared to PD mice. (**f**) Heatmap showing the top 20 increased and decreased metabolites (as indicated by Log_2_FC) in fecal extract from mice of HF group compared to mice of PD group. Significantly different metabolites were screened out by PLS-DA model (VIP > 1 and *p* < 0.05).

To assess the alteration in the microorganism metabolite upon silymarin and polyherbal extract supplement and further elucidate which metabolites might be involved in the improvement, liquid chromatography-mass spectrometry (LC-MS) non-targeted metabolomic analyses on the fecal samples obtained from HF, HSL, and PD groups were performed. Metabolites in a partial least squares-discriminant analysis (PLS-DA) model, which elucidated a high stability and predictability (Figure A3a,b), delineated a marked difference in the pairs of HF vs. HSL and HF vs. PD (Figure A3c,d). 246 metabolites were upregulated while 242 metabolites were downregulated in the HSL group (Figure 5a) and polyherbal extract resulted in the upregulation of 247 metabolites and downregulated of 288 metabolites (Figure A3e). As illustrated in Figure 5b, HSL intervention inhibited the HFD-induced enrichment of secondary BAs and derivatives (Figure 5c,d; Lithocholic acid, Taurolithocholic acid 3-sulfate, etc.), long-chain fatty acids (Arachidic acid, Palmitic acid, etc.), amino acids, and derivatives (Isoleucine, Indole-3-lactic acid, etc.) in the feces simultaneously with an elevated level of primary BAs (Figure 5e; Cholic acid, etc.), glycerophospholipids (Lysophosphatidylglycerol, etc.), prostaglandins (Prostaglandin E1, etc.), glutathione and derivatives, peptides (Gamma-Glu-Leu, Glu-Glu, etc.), and flavonoid. Moreover, polyherbal extract intervention likewise led to a reduction in secondary BAs and long-chain fatty acids, as well as a concurrent elevation in primary BAs and peptide levels (Figure A3f).

**Figure 6:**
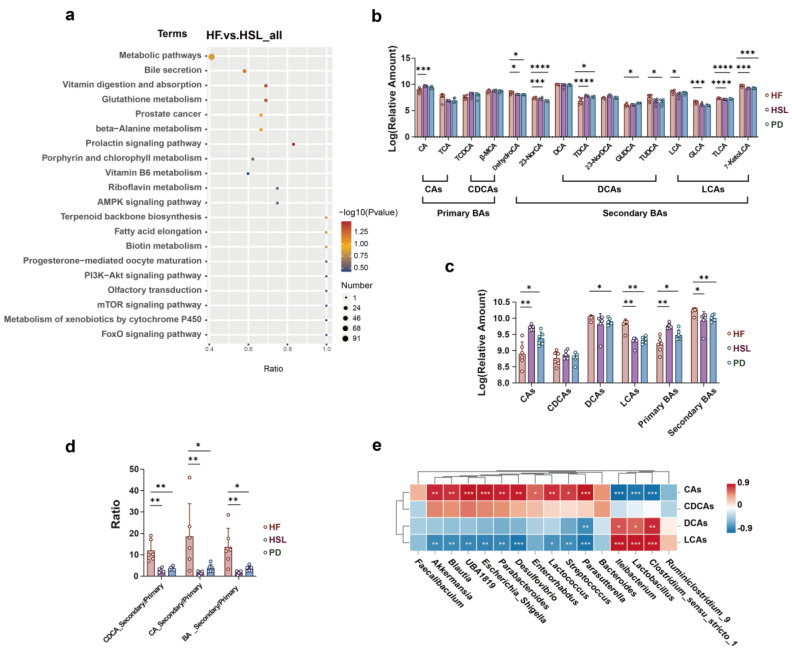
Effect of silymarin and polyherbal extract intervention on fecal BAs profile and its correlation with microbial community. (**a**) Enriched KEGG pathways in HF group compared with HSL group shows the top 20 regulated metabolic pathways by silymarin intervention. Rich Factor was determined by the ratio of differential metabolites detected to total metabolites in that pathway. (**b**) Relative quantitative value of various BAs in feces. (**c**,**d**) Diagram shows the significant differences in the level of classified CAs, CDCAs, DCAs, LCAs, classified primary BAs and secondary BAs (**c**), and the ratio of secondary BAs to primary BAs from different sources (**d**). (**e**) The correlations between the abundance of selected differentially abundant genus with the relative level of fecal CAs, CDCAs, DCAs, LCAs and in HF and HSL mice were analyzed by two-sided Spearman’s correlation. *n* = 6 individuals/group and data were represented as mean ± SD, the differences between groups were calculated by two-sided Mann–Whitney test; * *p* < 0.05, ** *p* < 0.01, *** *p* < 0.001, **** *p* < 0.0001. CAs cholic acid and conjugates, CDCAs chenodeoxycholic acid and conjugates, DCAs deoxycholic acid and conjugates, LCAs lithocholic acid and conjugates.

KEGG pathway enrichment analysis between HF and HSL groups depicted the pathway of bile secretion that was identified and marked (Figure 6a). Given the observed alterations in gut microbiota composition and differential metabolite analysis that both suggested a potential pathway related to BAs, we subsequently conducted inter-group comparisons on the relative quantification data of fecal BAs. The finding revealed that silymarin and polyherbal extract led to a reduction in taurolithocholic acid (TLCA) and 7-ketolithocholic acid (7-KetoLCA), while elevating the relative content of cholic acid (CA) (Figure 6b). Further categorizing BAs into primary and secondary BAs based on whether they were biotransformed form gut microbiota, it was unveiled that there was a decrease in secondary BAs and an increase in primary BAs following both silymarin and polyherbal extract intervention (Figure 6c), along with the markedly reduced ratio of secondary to primary BAs (Figure 6d). Consistently, Spearman correlation analysis identified significant positive correlations between altered *Ileibacterium*, *Lactobacillus*, *Clostridium,* and secondary BAs, and a significant negative correlation with primary BAs as CAs (Figure 6e). Confirmedly, the above genera were generally considered as the key bacteria of secondary BAs biosynthesis. The results indicated that silymarin and polyherbal extract reduced the levels of intestinal secondary bile acids, and the phenomenon was associated with the decrease in *Ileibacterium*, *Lactobacillus,* and *Clostridium* following treatment.

### 3.5. Silymarin and Polyherbal Extract Intervention Alter Serum BA Profiles

**Figure 7:**
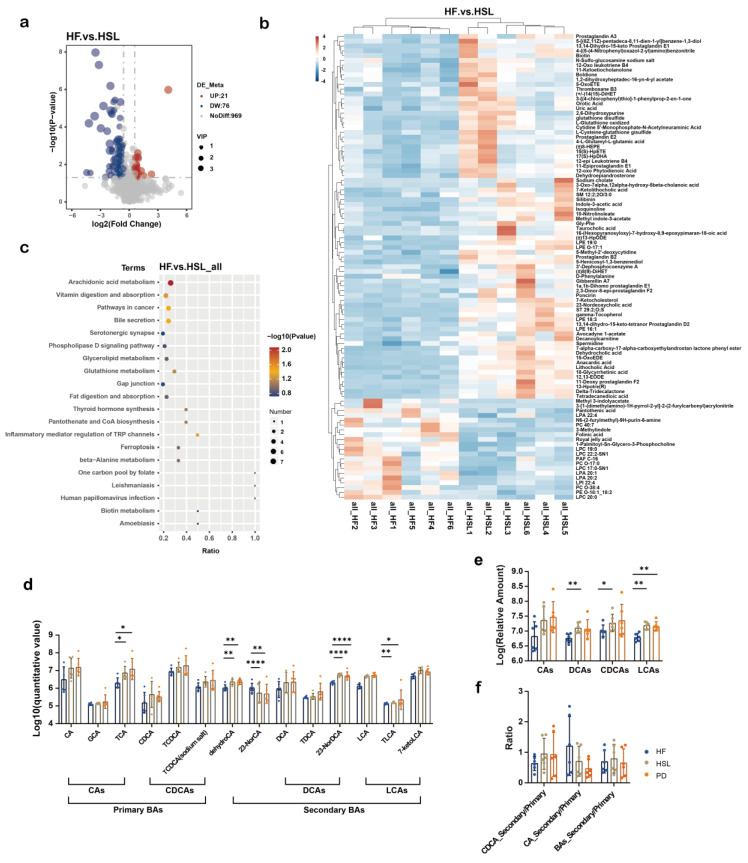
Alterations of metabolites in the serum of NAFLD mice upon silymarin intervention. (**a**) Volcano plot shows the number of significantly changed metabolites in the serum (determined by Log_2_ FC and −Log_10_ *p* value) of HF compared to HSL mice. (**b**) Significantly differed metabolites between the serum of HF group and HSL group, blocks in red and blue denote high and low FC values, respectively. (**c**) Enriched KEGG pathways in HF group compared with HSL group shows the top 20 regulated metabolic pathways by silymarin intervention; Rich Factor was determined by the ratio of differential metabolites detected to total metabolites in that pathway. (**d**–**f**) Diagram showing the significant differences among groups in the relative level of BAs (**d**), the classified CAs, CDCAs, LCAs, DCAs obtained from non-targeted metabonomics (**e**) and the ratio of secondary BAs to primary BAs from different sources (**f**); *n* = 6. Significantly different metabolites were screened out by established PLS-DA model (VIP > 1 and *p* < 0.05) and values were shown as mean ± SD while two-sided Mann–Whitney nonparametric test were conducted for comparisons. * *p* < 0.05, ** *p* < 0.01, **** *p* < 0.0001.

**Figure A4:**
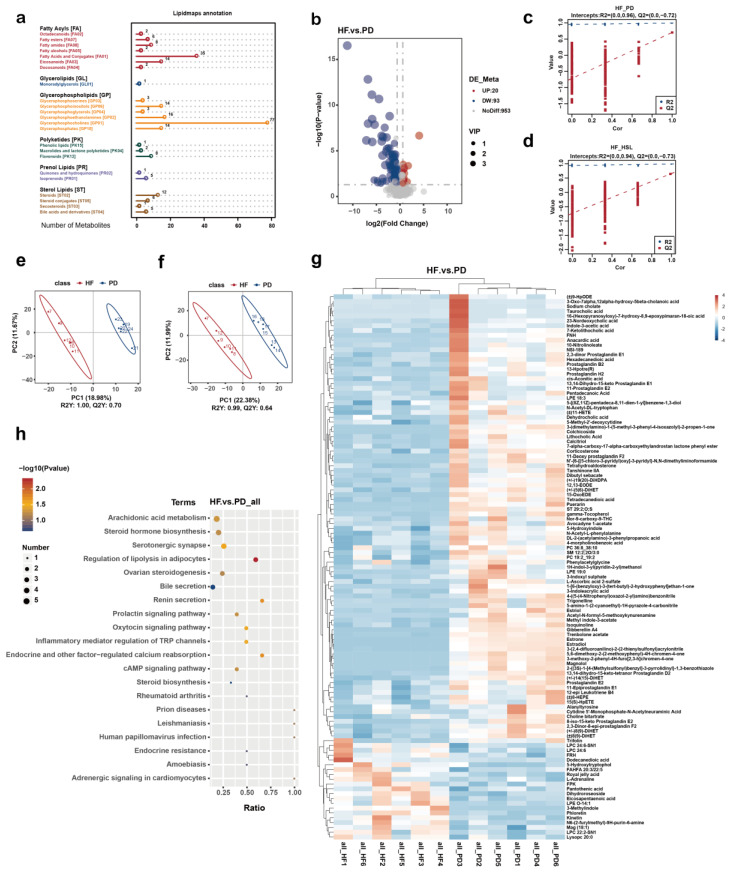
Alterations of metabolites in the serum of NAFLD mice upon polyherbal extract intervention. Analysis results of non-targeted metabolomics in the serum of HF, HSL and PD mice; *n* = 6. (**a**) Annotation of metabolite classification results based on LIPID MAPS database. (**b**) Volcano plot showing the number of upregulated and downregulated metabolites with significant differences in the serum of HF mice compared to PD mice. (**c**–**f**) Results of permutation tests conducted with 999 random per mutations in the PLS-DA model (**c**,**d**) and the PCA plot (**e**,**f**) of HF vs. HSL and HF vs. PD comparisons. (**g**) Markedly differed metabolites (as indicated by Log_2_FC) in the serum of HF group compared to PD group. (**h**) Enriched KEGG pathways in HF group compared with PD group shows the top 20 regulated metabolic pathways; Rich Factor was determined by the ratio of differential metabolites detected to total metabolites of serum samples in that pathway. Significantly different metabolites were screened out by PLS-DA model (VIP > 1 and *p* < 0.05).

To verify whether the above findings could reflect in the blood circulation, we subsequently proceeded with a non-targeted metabolomic analysis of the serum from NC, HF, HSL, and PD group. Metabolites were mainly annotated into Glycerophosphocholines based on a LIPID MAPS database (Figure A4a) and among the 1066 identified metabolites, 76 exhibited upregulation and 21 showed downregulation in response to silymarin intervention (Figure 7a). Additionally, 93 metabolites were upregulated and 20 were downregulated following polyherbal extract intervention (Figure A4b). Results from PLS-DA and permutation tests indicated robust model constructions for both the HF vs. HSL (Figure A4c,d) and HF vs. PD (Figure A4e,f) comparisons. On the serum of the HSL group, diminished levels of glycerophospholipids (Lysophosphatidylcholine, Lysophosphatidic acid) and 3-methylindole were observed, while an elevation in BAs and derivatives (Sodium cholate, Taurocholic acid), prostaglandins, glutathione and derivatives, and 3-acetate indole was noted (Figure 7b). These trends were aligned with the alterations investigated in the serum metabolites of the PD group (Figure A4g).

Notably, we also found those modulated metabolites were significantly linked to bile secretion pathway in HSL vs. HF (Figure 7c) and PD vs. HF (Figure A4h) comparisons through KEGG pathway analysis. Therefore, following the analysis approach of BAs in feces, we compared the levels of BAs in the serum (Figure 7d) and observed significant changes in BAs (Figure 7e), while the ratio of secondary to primary BAs differed without significance (Figure 7f). Results demonstrated a notable alteration in serum BAs profile following silymarin and polyherbal extract treatment, and further suggested that microbiota regulated BAs might be involved in the amelioration of NAFLD.

## 4. Discussion

**Figure 8:**
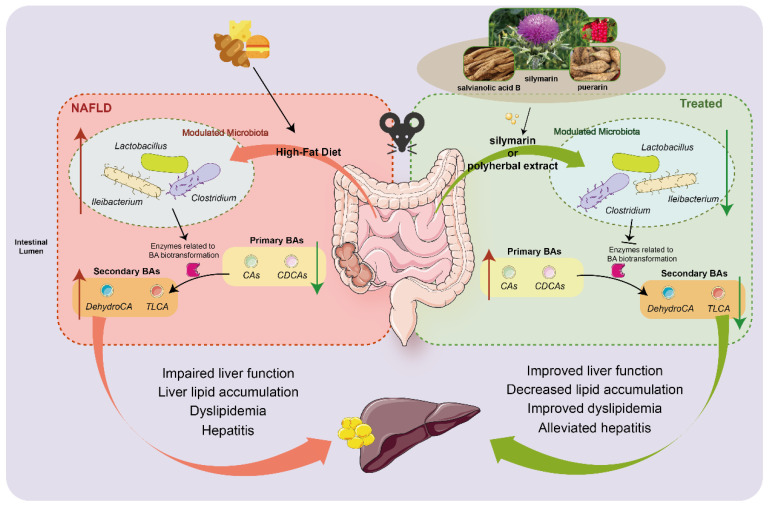
Schematic highlighting the primary findings of this study. Silymarin and polyherbal extract supplementation modulating the gut microbiota, significantly inhibiting the proliferation of Clostridium, Ileibacterium and Lactobacillus induced by high-fat diet, and further leading to a suppression in the synthesis of secondary BAs regulated by gut microbiota. The reduced levels of secondary BAs in the intestine and the improved BAs profile in blood circulation might be involved in the process of silymarin and polyherbal extract improving NAFLD.

Natural plant extracts have been a focal point of research for their potential as viable healthy food agents to ameliorate NAFLD. The present study demonstrated that silymarin or silymarin with salvianolic acids B and puerarin formula-improved HFD-induced hepatic steatosis. Furthermore, the beneficial alterations might be linked with gut microbiota and their metabolites, especially BAs as a crucial one for the amelioration of NAFLD (Figure 8).

Flavonoid compound silymarin has long been utilized in traditional medicine for treating liver and bile diseases [45]. Numerous recent studies have observed the positive effects of silymarin in improving NAFLD [46,47]. Several clinical randomized controlled trials indicate that silymarin contributes significantly to ameliorating the liver of patients with NAFLD. This improvement includes reductions in liver fat deposition, hepatocellular ballooning, and liver fibrosis, along with decreases in liver transaminase levels [26,48,49,50]. Similarly, several studies suggest that Sal B, a natural polyphenol compound derived from Radix Salvia Miltiorrhiza, exhibits protective effects against hepatic fat deposition and inflammation induced by an HFD [32,51,52]. Additionally, as a type of flavonoid compound, puerarin has also been extensively researched, demonstrating its potential to treat various chronic diseases, including NAFLD [33,53,54]. The present study provides robust evidence supporting the notion mentioned above. In lieu of the oral gavage method, we incorporated silymarin or polyherbal extract (silymarin in combination with Sal B and puerarin) into an HFD for the treatment of NAFLD in mice. We observed that both silymarin and polyherbal extract significantly improved NAFLD, as manifested by reduced hepatic lipid droplet accumulation, enhanced liver function, decreased levels of hepatic TG and serum TC, and the restoration of glucose tolerance. Additionally, insulin resistance, a factor associated with NAFLD, and levels of liver inflammatory cytokines TNF-α and IL-6, were also ameliorated with supplementation of silymarin.

Generally, insulin resistance is associated with lower HDL-C levels [55]. In this study, both the MSL and HSL groups showed improvements in insulin levels, yet the HDL-C levels in mice from these groups, as well as the PD group, decreased, which seems inconsistent with the aforementioned research. However, some studies have found that increases in HDL-C levels may also be accompanied by hepatic lipid accumulation and worsening insulin resistance [56,57]. Interestingly, following intervention with certain phytochemicals in mice, both increased HDL-C levels and improvements in insulin resistance have been observed [58,59]. The significant improvement in hepatic steatosis in this study may be related to more cholesterol being metabolized into bile acids in the liver. Therefore, a comprehensive consideration of hepatic lipid metabolism, serum HDL levels, and insulin resistance is needed to interpret the results. In addition, it is noteworthy that the weight change in treated mice was observed without significant differences compared to HFD mice, suggesting that the beneficial effects of silymarin might not be related to reduced energy intake.

Given the widely acknowledged dysregulation of the gut microbiota in the pathogenesis and progression of NAFLD, we examined whether the dysbiosis could be improved following silymarin and polyherbal extract intervention. At the genus level, supplementation with high doses of silymarin and polyherbal extract increased the abundance of probiotics like *Akkermansia* and *Blautia*. Moreover, silymarin and polyherbal extract resulted in the significant suppression of HFD-induced genera such as *Lactobacillus*, *Bacteroides*, *Clostridium*, and *Ileibacterium*. These bacteria above are reported to exhibit bile salt hydrolase activity and are primary contributors to the secondary BA synthesis [60,61]. Further, we observed that the transplantation of silymarin and polyherbal extract adapted feces likewise improved NAFLD, and changes in *Clostridium* and *Blautia* in the FMT experiment aligned with the results of the intervention experiment. The present findings validated that silymarin and polyherbal extract could enhance liver function and ameliorate NAFLD by modulating the gut microbiota.

The alteration of gut microbiota composition usually generated the different microbiota metabolites which are directly linked with the changes in hepatic pathogenesis [9,11]. We employed untargeted metabolomics to further explore the impact of gut microbiota on metabolite levels. As anticipated, the differential metabolites changed by silymarin and polyherbal extract intervention primarily include BAs and derivatives, carboxylic acids and their derivatives, glycerophospholipids, medium and long-chain fatty acids, glutathione and its derivatives, and branched-chain amino acids (BCAAs) and their derivatives.

The enriched *Akkermansia* and *Blautia* after intervention has been proven in other studies to produce short-chain fatty acids (SCFAs) that contribute to the NAFLD improvement [62,63,64,65,66]. SCFAs such as acetate and butyrate salts can significantly improve intestinal barrier damage through anti-inflammatory and antioxidant pathways [67,68], and regulate hepatic lipid synthesis, oxidation, and glucose homeostasis via adenosine monophosphate-activated protein kinase (AMPK)-dependent mechanisms [69,70]. Additionally, recent research suggests that butyrate can directly reduce intrahepatic pro-inflammatory cytokine release by modulating liver immune cells, and attenuate hepatic inflammation and oxidative damage by regulating the nuclear factor NF-E2-related antioxidant enzyme pathway [71,72]. Similarly, an increase in SCFA-related derivatives and an upregulation of β-amino acid metabolism in the feces and serum of mice were observed following the intervention, while β-amino acid reported being ultimately metabolized to acetate under normal conditions. The above evidence indicated that enhancing the production of SCFAs metabolized by gut microbiota might be one of the mechanisms through which silybin and polyherbal extract exert their effects in ameliorating NAFLD.

On another front, the enrichment of glutathione and its derivatives in feces and serum following intervention is proposed as a potential strategy for treating NAFLD due to their sulfur-containing moieties, which can reduce oxidative damage to hepatocyte, repair damaged hepatic cells, and promote its regeneration [73,74]. Additionally, elevated levels of BCAAs in the blood have been associated with metabolic syndromes such as NAFLD and type 2 diabetes in numerous clinical trials [75]. The present results showed that the increase in glycine and its downstream glutathione, and reduction in BCAAs such as isoleucine in serum and feces, may play a beneficial role in improving NAFLD with silymarin and polyherbal extract through the reshaped microbiota.

Previous studies have reported elevated levels of secondary BAs in the feces of mice fed with an HFD [76,77,78]. We found that intervention with silymarin and polyherbal extract led to a reduction in secondary BAs such as lithocholic acid, an increase in primary bile acid CA, and a decrease in the ratio of secondary to primary BAs in feces. These were consistent with the inhibition of microbiota involved in secondary BAs synthesis. Through correlation analysis, we further identified a positive correlation between intestinal *Clostridium*, *Lactobacillus*, *Ileibacterium*, and secondary BAs. Additionally, the serum levels of taurine-conjugated bile acids (taurocholic acid (TCA) and TLCA) increased following intervention with silymarin and polyherbal extract. Lei et al.’s study demonstrated that inhibition of *Clostridium*-mediated 7α-dehydroxylation suppressed secondary BAs biosynthesis, and the altered BAs profile further improve steatohepatitis [79]. Experiments involving lotus seed resistant starch supplementation also observed the inhibition of secondary BAs following the modulation of microbial composition exerted lipid-lowering effects [80]. These findings suggest that the modified composition of bile acids resulting from the reshaped microbiota could serve as one of the mechanisms through which silymarin and polyherbal extract manifest their favorable effects.

The BA pool is regulated by both the composition of the gut microbiota and the host’s BA transport and biosynthesis mechanisms. Intestinal bacteria catalyze the production of secondary BAs through bile salt hydrolysis and 7α-dehydroxylation [60]. The interaction between BAs and the gut microbiota significantly influences host metabolism [81,82]. Recent studies underscore the pivotal role of the gut microbiota-BA axis in NAFLD [83]. Extensive research indicates that the Farnesoid X Receptor (FXR) and the G protein-coupled receptor 5 (TGR5) are widely presented in the body, and serve as important targets for the biological activity of BAs in the enterohepatic circulation [84,85], with most secondary BAs and conjugated BAs acting as potent FXR agonists [81,86]. It is intriguing that numerous studies have provided evidence supporting the contrasting roles of hepatic FXR signaling and intestinal FXR signaling. Activation of hepatic FXR can mitigate hepatic lipid uptake and steatosis, whereas activation of intestinal FXR signaling is associated with disrupted cholesterol metabolism [87,88,89]. For instance, mice orally administered theabrownin exhibit inhibition of bacterial groups rich in bile salt hydrolase, leading to the accumulation of conjugated BAs in the distal ileum. This suppressed intestinal FXR and downstream signaling, while circulating conjugated BAs activated hepatic FXR, collectively contributes to improved lipid metabolism [90].

In the liver, activation of the FXR by agonists inhibits the expression of sterol regulatory element-binding protein 1c (SREBP-1c) via recombinant small heterodimer partner (SHP) pathway, and upregulates peroxisome proliferator-activated receptor alpha (PPARα), thereby increasing fatty acid β-oxidation and limiting hepatic lipid accumulation [91]. Independent of the SHP-SREBP1c pathway, FXR activation can also suppress the expression of key lipogenic genes, Scd1, Dgat2, and Lpin1, thereby reducing hepatic monounsaturated fatty acid synthesis [92]. Moreover, increased expression of TGR5 in the liver inhibits activation of the NLRP3 inflammasome and cleavage of caspase-1, thus alleviating inflammation and contributing to the improvement in NASH [93].

In the intestine, BAs can induce the expression of fibroblast growth factor 19 (FGF19) and fibroblast growth factor 15 (FGF15) upon FXR activation, releasing them into circulation. This activates the fibroblast growth factor receptor 4 (FGFR4) and beta-Klotho complex in the liver, inhibiting the expression of hepatic cholesterol synthesis enzymes CYP7A1 and CYP8B1. The above effects decrease cholesterol conversion and increase hepatic cholesterol levels, thereby exacerbating triglyceride accumulation [94,95,96,97]. Conversely, inhibition of intestinal FXR transcriptional activity following reduced secondary BAs may downregulate serum FGF15/FGF19 levels. This potentially upregulates the expression of key enzymes involved in BA synthesis in the liver, thus improving NAFLD by promoting cholesterol conversion [98]. Additionally, inhibiting the intestinal FXR-neuronal ceramide axis has been found to play a crucial role in preventing hepatic steatosis [85,99].

Based on the evidence presented, we speculated that the reduction in secondary BAs in the gut might ameliorate disruptions of cholesterol metabolism in the liver induced by intestinal FXR activation; meanwhile, the enriched spectrum of conjugated bile acids in the serum could stimulate hepatic FXR and TGR5 receptors, thereby reducing hepatic lipid accumulation through various mechanisms such as decreased synthesis and oxidation of fatty acids and triglycerides. The alteration in the profile of BAs, both at the intestinal and circulatory levels, could thus improve hepatic steatosis and confer a protective effect on the liver. Another possibility is that the absorption of dietary lipids in the intestine requires triglycerides to be dispersed in micelles containing BAs and phospholipids and changes in intestinal BAs may affect the lipid absorption process [100]. For example, in HFD mice, certain BAs (as agonists) can significantly stimulate the intestinal FXR-SR-B1 pathway to increase chylomicron secretion and lead to the excessive utilization of fats [17]. In our study, silymarin and polyherbal extract resulted in the accumulation of lipid-acyl compounds and glycerophospholipids in feces, suggesting that the reduction in secondary BAs in feces might also ameliorate NAFLD by suppressing the release of chylomicrons and further inhibiting excessive lipid absorption and utilization of the intestinal tract.

However, our results are also subject to bias because we only conducted this intervention in mice, which differ from humans genetically, physiologically, and environmentally. The intervention dosage used has not been validated in human trials. Additionally, there is no specific evidence for the exact microbiota and metabolites involved in NAFLD improvement by silymarin and polyphenol extracts in humans. Therefore, caution should be exercised when extrapolating the results to humanity.

## 5. Conclusions

Our study reveals that the intervention of silymarin alone or in combination with Sal B and puerarin significantly alters the intestinal microbial composition in NAFLD mice, which is reflected in the increase of beneficial probiotics such as *Akkermansia* and *Blautia*, and the suppression of the genera related to secondary BAs synthesis such as *Clostridium* and *Bacteroides*. Results from the FMT confirmed that the alteration of microbiota and its metabolites was a crucial link in silymarin’s ability to reduce hepatic lipid accumulation, enhance liver function, and improve NAFLD. Additionally, the silymarin-regulated microbiota significantly inhibited the synthesis of intestinal secondary BAs, and TCA and TLCA were enriched in the serum. The present findings provide a scientific basis for the further exploration of the mechanisms underlying the impact of altered BAs profile influenced by intestinal microbiota on improving NAFLD.

## Figures and Tables

**Table 1:** The substance content of 100.3 g intervention feeds.

Ingredient	HF	MSL	HSL	PD
Silybin (g)	-	0.101	0.202	0.101
Sal B (g)	-	-	-	0.046
Puerarin (g)	-	-	-	0.042
Fat (g)	24.000	24.000	24.000	24.000
Protein (g)	24.000	24.000	24.000	24.000
Carbohydrate (g)	41.000	41.000	41.000	41.000
Microelement (g)	11.000	11.000	11.000	11.000
Sterile water (g)	0.3	0.199	0.098	0.111

## Data Availability

We state that data, analytic methods, and study materials will be made available to other researchers, upon request. The raw data for 16S rRNA sequencing in this study are publicly available online: https://www.ncbi.nlm.nih.gov/bioproject/PRJNA1086335 (accessed on 11 March 2024) and https://www.ncbi.nlm.nih.gov/bioproject/PRJNA1086349 (accessed on 11 March 2024).
